# Isofuranodiene, a Natural Sesquiterpene Isolated from Wild Celery (*Smyrnium olusatrum* L.), Protects Rats against Acute Ischemic Stroke

**DOI:** 10.3390/ph14040344

**Published:** 2021-04-09

**Authors:** Hasan Yousefi-Manesh, Ahmad Reza Dehpour, Samira Shirooie, Fariba Bagheri, Vida Farrokhi, Seyyedeh Elaheh Mousavi, Massimo Ricciutelli, Loredana Cappellacci, Víctor López, Filippo Maggi, Riccardo Petrelli

**Affiliations:** 1 Department of Pharmacology, School of Medicine, Tehran University of Medical Sciences, Tehran 1417613151, Iran; hasanyousefimanesh@gmail.com (H.Y.-M.); dehpour@yahoo.com (A.R.D.); f.bagheri.3223@gmail.com (F.B.); vida.farrokhi@yahoo.com (V.F.); semousavi@sina.tums.ac.ir (S.E.M.); 2 Experimental Medicine Research Center, Tehran University of Medical Sciences, Tehran 1417613151, Iran; 3 Pharmaceutical Sciences Research Center, Health Institute, Kermanshah University of Medical Sciences, Kermanshah 6734667149, Iran; 4 School of Pharmacy, University of Camerino, 62032 Camerino, Italy; massimo.ricciutelli@unicam.it (M.R.); loredana.cappellacci@unicam.it (L.C.); riccardo.petrelli@unicam.it (R.P.); 5 Department of Pharmacy, Faculty of Health Sciences, Universidad San Jorge, Villanueva de Gállego, 50830 Zaragoza, Spain; ilopez@usj.es

**Keywords:** isofuranodiene, *Smyrnium olusatrum* L., ischemic stroke, NF-ĸB, IL-1β, TNF-α

## Abstract

The myrrh-like furanosesquiterpene isofuranodiene (IFD) is the main constituent of wild celery (*Smyrnium olusatrum* L., Apiaceae), an overlooked vegetable that was cultivated during the Roman Empire. In the present study, we investigated the protective effects of IFD pre-treatment against oxidative stress and inflammatory response in an animal model of ischemic stroke. IFD was isolated by the crystallization of *Smyrnium olusatrum* essential oil, and its structure and purity were confirmed by NMR and HPLC analyses. Acute pre-treatment of IFD (10 mg/kg i.p.) significantly reduced the levels of the inflammatory cytokines IL-1β and TNF-α, the expression of pNF-κB/NF-κB, and the lipid peroxidation indicator MDA. Finally, IFD boosted a faster recovery and better scores in grid-walking and modified neurological severity scores (mNSS) tests. Taken together, these findings indicate IFD as a promising lead compound for the discovery of new treatments of brain ischemia.

## 1. Introduction

Globalization has led to a change in our eating habits and the disappearance of many plants that were once eaten by older generations. Among them, the aromatic herb *Smyrnium olusatrum* L. (Apiaceae), also known as Alexanders or wild celery, represents an interesting case. *Smyrnium olusatrum* L. is a biennial plant belonging to the Apiaceae family, and is widespread in the Mediterranean basin, reaching the North African coast to the South and the British Isles to the North. It is found in shady places such as hedgerows, cliffs, and other uncultivated places, from sea level to high hills, often as a relic of kitchen gardens and monasteries. During the medieval period, *S. olusatrum* had been used as an antiscorbutic, stomachic, antiasthmatic, diuretic, and laxative remedy [1,2]. As a vegetable, *S. olusatrum* had been used until the medieval period, when it was replaced by common celery (*Apium graveolens* L.) due to changing tastes favoring tender and sweeter dishes instead of more spicy and hot ones [2]. *Smyrnium olusatrum* is a rich source of volatile sesquiterpenes containing a furan ring, among which isofuranodiene (syn. furanodiene, C_15_H_20_O, IFD) is the most abundant [3]. This compound is also a key marker of other medicinal and aromatic plant species such as *Commiphora myrrha* (Nees) Engl., *Curcuma wenyujin* Y.H. Chen and C. Ling, *Eugenia uniflora* L., *Chloranthus japonicus* Siebold and *Vepris unifoliolata* Baill.) Labat [1,3]. It has also been found in some marine organisms, such as corals [4]. It is noteworthy that our group has recently proven that IFD may exert neuritogenic, anti-inflammatory, and anticancer activities, as well as insecticidal and acaricidal properties [5,6,7,8,9]. In recent years, researchers’ increasing interest towards the protective role of natural substances, especially those contained in fruits, vegetables, and medicinal plants, against ischemic stroke has been observed. Thus, natural products are playing a notable role in discovering new therapeutic treatments for stroke.

Ischemic stroke is one of the most complicated, destructive, and fatal neurological disorders [10]. This disease is the second highest cause of death, and the third most common factor of disability worldwide [11]. From an economic perspective, stroke has a burden of approximately GBP 2.4 billion for informal care costs and GBP 2.1 billion for indirect costs such as benefit payments and productivity reduction, because of death and disability within the global health economy [11]. One of the most critical roles in the pathogenesis of ischemic stroke is played by inflammatory processes [12]. These processes will raise the amount of different inflammatory factors in several parts of the central nervous system (CNS), especially in the brain. During the reperfusion phase, which occurs after stroke alleviation, the production of reactive oxygen species (ROS) starts to increase due to hypoxia caused by disruption of the brain blood flow [13]. ROS generation can lead to overexpression of cell survival programs that relieve tissue injury caused by ischemia. However, this overexpression may cause oxidative stress that can advance to cell apoptosis and death [14]. The brain has a high concentration of peroxidizable lipids; therefore, it can be a suitable target for reactive oxygen species. Malondialdehyde (MDA), as the final product of lipid peroxidation, can cause cell toxicity and death. The amount of MDA produced is one of the most important biomarkers for measuring the brain’s oxidative stress level [15]. TNF-α and IL-1β progress the inflammatory response by releasing Essential Amino Acids (EAA), nitric oxide (NO), and free oxygen radicals, and increase stroke-induced tissue injury [16]. NF-κB is another factor that overexpresses after ischemic-reperfusion occurs in stroke. NF-κB causes tissue damage, apoptosis, and neuronal death in some brain regions such as the hippocampus, and triggers many inflammatory pathways and mediators [17]. Strokes may cause physical and behavioral disabilities, such as motor impairments. Most of these complications are caused by neuronal and tissue injury in the brain cortex’s motor regions and damage in subcortical projection pathways. Therefore, strokes may lead to a reduction in movement speed, ability, and control [18].

In the present work, we evaluated for the first time the neuroprotective effects of IFD against oxidative stress and inflammatory conditions in a rat model of ischemic stroke. For this purpose, the efficacy of IFD against bilateral carotid artery (BCA) occlusion injury was determined by evaluating the levels of pro-inflammatory cytokines (IL-1β and TNF-α) and MDA, a marker of lipid peroxidation, and the expression of pNF-κB/NF-κB in the rat brain. In addition, the effect of IFD treatment on rat behavior after brain stroke induction was determined by the grid-walking and modified neurological severity scores (mNSS) tests.

## 2. Results

### 2.1. IFD Shortened the Behavioral Recovery Period after the Brain Stroke

**Figure 1:**
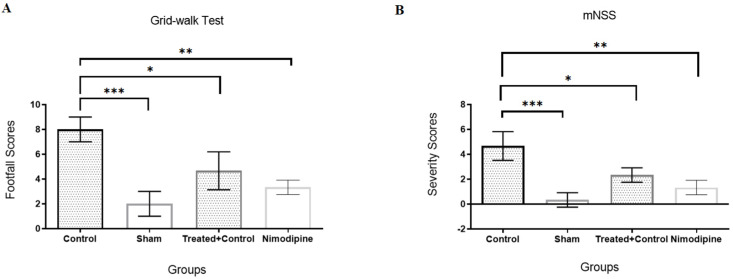
Behavioral tests in rats. (**A**): Grid-walking test, (**B**): modified neurological severity scores (mNSS) test. Data are mean ± SEM. * *p* < 0.05, ** *p* < 0.01, *** *p* < 0.001.

Two behavioral tests, namely mNSS and grid-walking, have been performed to evaluate rat neurological functioning after ischemic stroke. Statistically significant differences were observed in the grid-walking test and motor scores in the mNSS test between the IFD-treated rats and the non-treated stroke group (the control group) (*p* < 0.05). As shown in Figure 1A,B, IFD pre-treated rats had a faster recovery and better scores in the grid-walking and mNSS tests, whereas rats from the control group that received no treatment showed a lower rate of amelioration and higher scores in both tests compared with the sham group (*p* < 0.01). Moreover, there are no significant differences between the pre-treated group and the positive control group. It was noticed from this test that IFD improved the outcomes on the brain ischemic reperfused group.

### 2.2. Effects of IFD on Inflammatory Cytokines and Oxidative Stress in Ischemic Brains

**Figure 2:**
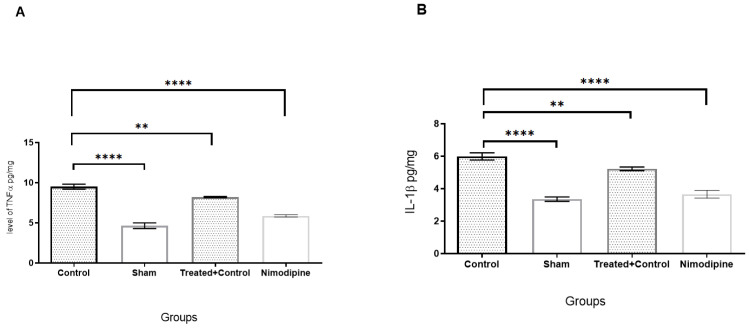

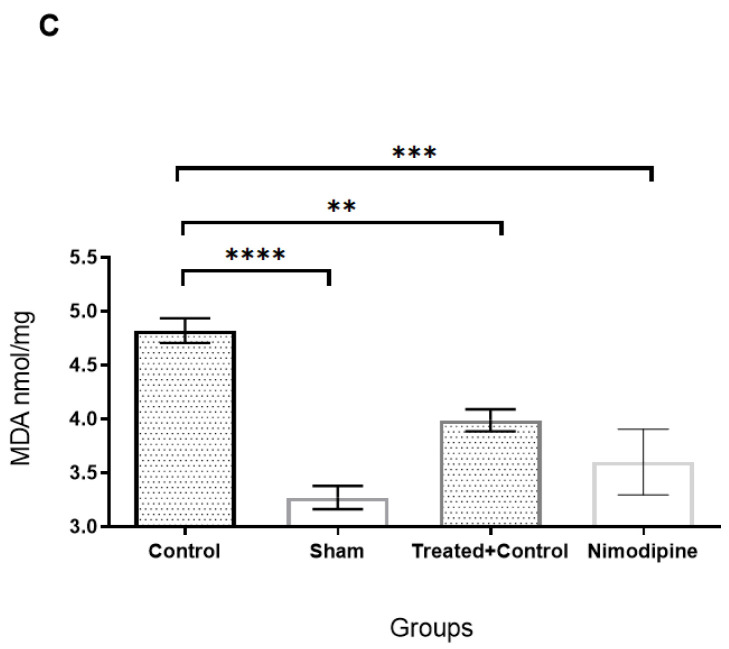
IFD pre-treatment reduced inflammatory cytokines and oxidative stress 24 h after ischemic stroke. (**A**): TNF-α, (**B**): IL-1β and (**C**): MDA levels of the brain tissues of the sham, control, IFD treatment + ischemic stroke, and Nimodipine as a positive control. All values are shown as mean ± SEM. ** *p* < 0.01, *** *p* < 0.001, **** *p* < 0.0001.

As shown in Figure 2A–C, 24 h after BCA occlusion, pro-inflammatory cytokines such as TNF-α and IL-1β, and MDA, as a lipid peroxidation marker, were assessed via ELISA assay. BCA occlusion (control group) showed increased levels of TNF-α, IL-1β, and MDA when compared with the sham group (*p* < 0.001). On the other hand, pre-treatment with IFD reversed these effects significantly compared to the control group (*p* < 0.01 and *p* < 0.001, respectively). In addition, MDA in the pre-treated group and the positive control group did not have a significant difference.

### 2.3. The Anti-Inflammatory Effects of IFD May Be Mediated through pNF-κB Protein Downregulation

**Figure 3:**
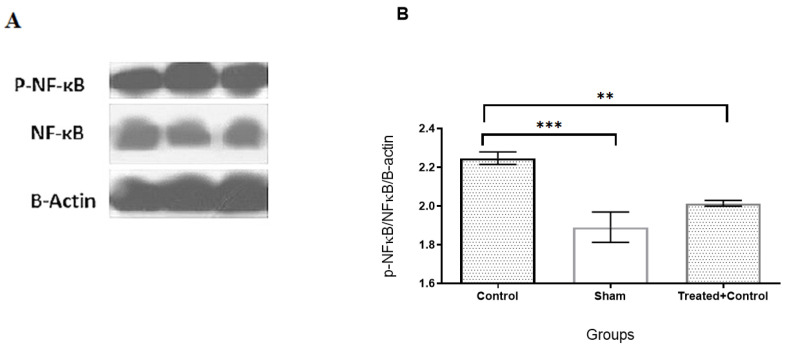
(**A**). Western blot of NF-κB phosphorylation in brain tissues of groups. (**B**). IFD pre-treatment reduced phosphorylation of NF-κB in animals 24 h after reperfusion. Data are shown as mean ± SEM. ** *p* < 0.01, *** *p* < 0.001.

The results of the Western blot analysis showed that BCA occlusion increased the phosphorylation of NF-κB p65 (active form) in the control group when compared with the sham group (*p* < 0.001). Of note, pre-treatment with IFD reduced its phosphorylated form significantly in respect to the control group (*p* < 0.01) (Figure 3A,B).

## 3. Discussion

IFD has attracted the attention of many researchers and academics in recent years due to its anticancer, analgesic, neuritogenic, anti-inflammatory, hepatoprotective, antiprotozoal, and insecticidal properties [5,7,8,19,20,21,22].

In the present study, we highlighted for the first time the neuroprotective effects of IFD on an acutely pre-treated ischemic stroke rat model by measuring biochemical markers, pathology of the hippocampi cells, and behavioral assays. Li et al. have indicated that IFD has anti-inflammatory effects on liver injury induced by d-galactosamine/lipopolysaccharide in rats, by reducing inflammatory cytokines such as IL6, IL-1*β*, and TNF-*α*, decreasing the marker of peroxidation of lipids, MDA, and providing hepatoprotection by a reduction in aminotransferase levels [6]. Previous research has indicated that compounds with a similar structure to IFD prevented inflammation and reduced the expression of pro-inflammatory cytokines [23,24]. Owing to its hydrophobic nature and small size, IFD can cross the blood–brain barrier, exerting its effect in neurons [5]. This effect enhances the neuroprotective potential of IFD, making it valuable for the treatment of brain disorders.

According to the biochemical assessments in our study, the acute pre-treatment with IFD significantly reduced the levels of the inflammatory cytokines IL-1*β* and TNF-α, which are both important mediators of neuroinflammation in ischemic stroke, and decreased the lipid peroxidation indicator MDA, as a marker of oxidative stress. In addition, IFD ameliorated the behavioral effects, improving the sensorimotor reflexes and reducing the injury’s score severity. NF-κB has an important role in impairment during brain ischemia. For example, hypoxia, ROS, and other inflammatory mediators activate NF-kB signaling, increase phosphorylation, and activate the NF-κB p65 subunit [25]. During cerebral ischemic-reperfusion, NF-κB is involved in many processes, e.g., apoptosis and inflammation, by regulating gene expression [26]. NF-κB hyperactivity, as an initial factor of inflammation in the brain, leads to apoptosis of the hippocampus neurons and elevates cytokine expressions such as IL-6, IL-8, and TNF-α in neurons, endothelial and glial cells [27]. In addition, NF-κB increases the expression of pro-apoptotic genes such as P53, iNOS, and COX-2 in glial and neuronal cells [28]. Our findings showed that pre-treatment with IFD reduced phosphorylation and activation of NF-κB in the rat brains’ prefrontal cortex compared with the control group. This result supports the hypothesis that IFD is able to prevent the inflammatory cascade and apoptosis of neurons. These results are partly supported by our previous study in which IFD was found to mimic the neuroactivity of NGF for neuronal survival, development, and differentiation [5].

It is worth mentioning that IFD is present in high quantities in all the parts of *S. olusatrum*, and it can be easily collected in the EO obtained by hydrodistillation. Wild celery represents today an ideal and rich source for the extraction of IFD. In this regard, Maggi et al. led an intense campaign to isolate EO from roots, basal leaves, flowers, and green or ripe fruits of wild celery, highlighting high IFD levels in different plant organs [1,2,3]. In detail, EO from basal leaves contained up to 37.2% IFD, the EO from roots contained up to 46.6% IFD, whereas the EO obtained from fruits (green and ripe) contained up to 31.5% and 20.7% IFD, respectively. Lastly, the EO from the flowers contained a maximum of 56.2% IFD. This research has laid the foundations for IFD extraction’s scalability from *S. olusatrum*, highlighting the flowers as the best candidate due to the high concentration of the target molecule. The isolation and purification of a bulk amount of IFD can be easily carried out by crystallization or, alternatively, flash chromatography [5].

## 4. Materials and Methods

### 4.1. Purification and Analysis of IFD

**Figure 4:**
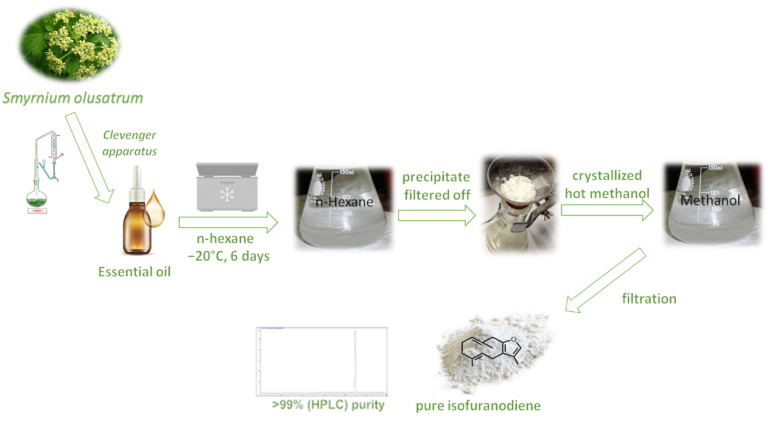
Isolation and purification of isofuranodiene.

The plant material (inflorescences) used was from a wild population of *S. olusatrum* growing in Pioraco, central Italy (N 43°10′41″; E 12°59′27″, 440 m a.s.l.), collected in April 2018. A herbarium specimen was deposited in the *Herbarium Camerinensis* of the University of Camerino, under the codex CAME 25677. Fresh inflorescences were hydrodistilled for 4 h using a Clevenger-type apparatus in order to obtain a yellowish essential oil with a yield of 1.8% on a dry weight basis. Afterward, crude crystals of IFD (C_15_H_20_O) were obtained by adding analytical grade hexane (300 mL) to 10 g of flower essential oil and storing the mixture at –20 °C for 6 days. Once all crude crystals had been filtered out, they were recrystallized (3 times) using hot methanol, obtaining pure white crystals of IFD (yield = 75%) (Figure 1). Accurate 1D (^1^H NMR and ^13^C NMR) and 2D (COSY, TOCSY, and HMBC) NMR studies were carried out on a Bruker Avance III 500 MHz spectrometer (Billerica, MA), and comparison with the data reported in the literature helped us to confirm the IFD’s structure [7,22]. The purity of IFD (~99%) was assessed by HPLC using an HP-1100 series (Agilent Technologies, Palo Alto, CA, USA) LC system equipped with a diode array detector (DAD). The separation was accomplished on a Kinetex© PFP (100A, 100 × 4.6 mm i.d., 2.6 μm), thermostatted at 40 °C using H_2_O (Milli-Q SP Reagent Water System, Millipore, Bedford, MA, USA) and CH_3_CN (Carlo Erba Acetonitrile for HPLC Plus gradient, Milan, Italy) as mobile phases A and B, respectively. The gradient elution (1.0 mL/min) was set as follows: 0–15 min (40% B), 15–30 min (60% B). IFD was diluted in CH_3_CN and injected (5 μL) into HPLC using disposable Minisart SRP4 filters, with a pore width of 0.45 μm (Chromafil PET-20/25, Sartorius Stedim Biotech GmbH, Goettingen, Germany). The peak of IFD eluted at a retention time of 22.432 min (Figure 1) and was monitored at different wavelengths (220, 230, and 254 nm). The presence of curzerene, an artifact of IFD [1], has been excluded (Figure 4).

### 4.2. Animals and Experimental Groups

Twenty-eight male adult Wistar rats (8–10 weeks old) weighing 220–260 g were obtained from the Animal Center in Tehran University, Department of Pharmacology. Rats were maintained under standard laboratory conditions (temperature: 24 ± 1 °C, humidity: 60–65%, light/dark cycle: 12 h) with free access to standard animal food and water. All procedures were conducted in accordance with the guidelines of the Animal Ethics Committee of Tehran University of Medical Sciences (ethical approval number is 989843). They also complied with the NIH guide for the care and use of laboratory animals (NIH Publication No. 80-23; revised 1978).

The rats were randomly divided into four groups:

1. Sham group (saline injection as vehicle and surgery without induction of BCA occlusion and treatment).
2. Control group (saline injection as vehicle and induction of BCA occlusion without treatment).
3. Treated + control group (pre-administration of IFD + BCA occlusion model induction).
4. Positive control group (pre-administration of Nimodipine + BCA occlusion model induction).

### 4.3. Induction of Ischemic Stroke

The rats were anesthetized with an intraperitoneal injection of Ketamine (45 mg/kg); then, after incision of the rat neck, carotid arteries were occluded by a clamp. After 30 min occlusion, the clamp was removed and 60 min reperfusion was performed [12].

### 4.4. Treatments

The number of rats in each group was 7. Rats in treated groups received a single dose of 10 mg/kg of IFD (dissolved in saline as a vehicle, i.p.). This dose was chosen according to the minimum dose with high efficacy. This dose was revealed to be safe, as shown in a previous study [6]. Rats in the positive control group received a single dose of 10 mg/kg of Nimodipine (i.p.) for 30 min before BCA occlusion induction.

### 4.5. Behavioral Tests

Twenty-four hours after brain stroke induction, the grid-walking and modified neurological severity scores (mNSS) tests were used to assess the behavioral deficiency and remission caused by IFD treatment in rats. For the grid-walking test, a grid floor (45 × 45 cm) with holes measuring 1 cm across and 2.5 cm in height was used, and an assessment of the modified neurological severity score was performed. Our scoring system was based on the animals’ footfall. The animals were allowed to walk freely on the grid floor for 1 min, and then video recording was started and analyzed by blind researchers. Each time the rat’s claws touched the floor it was scored two, and every time they pulled their paw before touching the floor it was scored one. Higher scores indicated more severe injuries [29]. The mNSS test evaluates the function of motor and sensory reflexes. For each inability to carry out the test, one point was scored. The scale was graded from 0 (normal function) to 18 (maximum inability) [30].

### 4.6. Molecular Assays

The whole-brain homogenates were centrifuged at 12,000 rpm for 15 min, and the supernatants were assessed for amounts of inflammatory mediators such as TNFα (no. CSB-E11987r) and IL-1β (no. CSB-E08055r) using an R&D systems ELISA kit. In addition, the amount of MDA in the homogenates was evaluated using the lipid peroxidation assay with an MDA assay kit (no. KA3736).

### 4.7. Western Blotting

The changes in the expression of pNF-ĸB/NF-ĸB in the prefrontal cortex of the rat brain were evaluated by the Western blot assay. Following centrifuging at 12,000 rpm for 20 min at 4 °C, 10 mg of protein was determined on 10% SDS-PAGE gel and transferred onto polyvinylidene difluoride (PVDF) membranes (Millipore, Germany). Five percent non-fat skim milk was used in order to obstruct membranes in 120 min. Then, membranes were incubated overnight with these primary antibodies: β-actin, NF-ĸB, and pNF-ĸB. All of the antibodies were purchased from Santa Cruz Biotechnology (Santa Cruz, CA, USA). Tris-Buffered Saline with Tween 20 (TBST) was used for washing the membranes 3 times; then, they were incubated with secondary antibodies at room temperature for 1 h. The BM chemiluminescence Western blot kit (Roche, Mannheim, Germany) was used to detect bands, whereas the software ImageJ (Version 1.52t, Wayne Rasband (NIH), Bethesda, MD, USA) was used to evaluate their optimal density. The relative amount of pNF-ĸB/NF-ĸB was determined using GraphPad Prism 6 (San Diego, CA, USA).

### 4.8. Statistical Analysis

In this study, all analyses were carried out with Graph Pad Prism version 9 and SPSS software (version 22) (SPSS Inc., Chicago, IL, USA). One-way analysis of variance (ANOVA) followed by Tukey’s post hoc test was used to evaluate multiple groups’ differences. A *p*-value less than 0.05 was determined as a statistically significant level.

## 5. Conclusions

In conclusion, IFD, a natural sesquiterpene obtainable in great amounts from wild celery by a scalable procedure, exerts protective effects against brain ischemia by suppressing NF-κB-activation and reducing pro-inflammatory cytokines. Our data demonstrate the neuroprotective properties of IFD, suggesting that it may be effective in stroke therapy. Moreover, IFD may be considered a lead compound for discovering new treatments of brain ischemia. However, further pre-clinical studies such as pharmacokinetics and toxicological studies are needed in order to confirm the use of IFD as a promising compound to manage severe problems in ischemic stroke.

## Data Availability

The data supporting the findings of this study are available on request from the corresponding authors upon reasonable request.
